# Psychometric Evaluation of the Whiteley Index-8 in Chinese Outpatients in General Hospitals

**DOI:** 10.3389/fpsyg.2021.557662

**Published:** 2021-07-01

**Authors:** Yixiao Chen, Per Fink, Jing Wei, Anne-Kristin Toussaint, Lan Zhang, Yaoyin Zhang, Hua Chen, Xiquan Ma, Wentian Li, Jie Ren, Wei Lu, Rainer Leonhart, Kurt Fritzsche, Heng Wu

**Affiliations:** ^1^Department of Psychosomatic Medicine, Shanghai Tongji Hospital, Tongji University School of Medicine, Shanghai, China; ^2^The Research Clinic for Functional Disorders and Psychosomatics, Aarhus University Hospital, Aarhus University, Aarhus, Denmark; ^3^Department of Psychological Medicine, Peking Union Medical College Hospital, Chinese Academy of Medical Sciences & Peking Union Medical College, Beijing, China; ^4^Department of Psychosomatic Medicine and Psychotherapy, University Medical Center Hamburg-Eppendorf, Hamburg, Germany; ^5^Mental Health Centre, West China Hospital, Sichuan University, Chengdu, China; ^6^Department of Psychosomatic Medicine, Sichuan Provincial People's Hospital, University of Electronic Science and Technology of China, Chengdu, China; ^7^Department of Psychological Medicine, Zhong Shan Hospital, Fudan University, Shanghai, China; ^8^Department of Psychosomatic Medicine, School of Medicine, Dongfang Hospital, Tongji University, Shanghai, China; ^9^Department of Clinical Psychology, Wuhan Mental Health Center, Wuhan, China; ^10^Department of Rehabilitation, General Hospital of Jincheng Anthracite Coal Mining Group Co. Ltd., Jincheng, China; ^11^Department of Psychosomatic Medicine, Beijing Hospital of Traditional Chinese Medicine, Capital University, Beijing, China; ^12^Institute of Psychology, University of Freiburg, Freiburg, Germany; ^13^Department of Psychosomatic Medicine and Psychotherapy, Faculty of Medicine, Medical Center – University of Freiburg, Freiburg, Germany

**Keywords:** Whiteley Index, health anxiety, reliability, validity, ROC

## Abstract

**Background:** Excessive and persistent health anxiety is a common and disabling but often unrecognized illness. Therefore, screening patients for health anxiety is recommended in primary care. The aim of the present study was to examine the psychometric properties of an updated version of the eight-item Whiteley Index (WI-8) among outpatients in general hospitals in China.

**Methods:** The presented data were derived from a multicenter cross-sectional study. The Chinese version of the WI-8 was administered to a total of 696 outpatients. Cronbach's alpha was used to evaluate the internal consistency of the scale. The validity of the scale was evaluated based on factor analysis and correlation analyses. To assess the discriminant ability, receiver operating characteristic (ROC) analysis was conducted.

**Results:** Cronbach's alpha was 0.937, and it decreased (0.925) after deleting the new 8th item. Factor analysis extracted one factor accounting for 69.2% of the variance. Moderate correlations were found (0.414–0.662) between the WI-8 and General Anxiety Disorder (GAD-7), Patient Health Questionnaire-9 (PHQ-9), Patient Health Questionnaire-15 (PHQ-15) and Somatic Symptom Disorder B-criteria (SSD-12). The ROC curve indicated excellent discriminatory ability to discriminate among patients with health anxiety (AUC = 0.822).

**Conclusions:** The new WI-8 version is a reliable and valid tool to screen for health anxiety in general hospital patients. We recommend the WI-8 as a useful screening tool for health anxiety.

## Background

Health anxiety is defined by cognitive-behavioral researchers as worry about health ranging from mild concern to excessive preoccupation (Ferguson, 2009; Longley et al., 2010). Excessive and persistent health anxiety is a common and disabling condition that can result in substantial suffering, difficult doctor-patient relationships and high health care costs (Robbins and Kirmayer, 1996; Barsky et al., 2001; Fink et al., 2010; Sunderland et al., 2013; Bobevski et al., 2016). Moreover, health anxiety can become chronic and incapacitating, with a majority (>60%) of cases still showing symptoms after several years of follow-up (Sadock, 2017). Health anxiety often remains unrecognized (Gureje et al., 1997; Conradt et al., 2006); thus, early detection is crucial for reducing such serious impairment. Screening for health anxiety among primary care patients has been proposed (Fink et al., 1999).

Health anxiety has often been referred to as hypochondriasis (American Psychiatric Association., 2000). However, the DSM-IV definition of hypochondriasis has received criticism because the criteria were too narrow to be applied in clinical practice (Fink et al., 2004). Therefore, the DSM-V replaced hypochondriasis, among others, with illness anxiety disorder (IAD) and somatic symptom disorder (SSD) (American Psychiatric Association, 2013), and the classifications of IAD and SSD have proven to be more reliable in detecting health anxiety than hypochondriasis (Newby et al., 2017). The difference between the two disorders lies in the severity of somatic symptoms. SSD diagnosis requires distressing and disabling somatic symptoms in criterion A, and related thoughts, feelings, and behaviors in criterion B. IAD is characterized by non-existent or relatively minor somatic symptoms, excessive fears of illness, and high levels of health anxiety (Sadock, 2017). Bailer et al. (2016) found no difference in health anxiety severity, other hypochondriacial characteristics, illness behavior, somatic symptom attributions, and physical concerns between IAD patients and SSD patients, whereas Newby et al. (2017) proposed that SSD patients show more severe health anxiety. Nevertheless, these studies have suggested that health anxiety is an important feature of IAD and SSD.

The Whiteley Index (WI) is a “classic” scale of hypochondria, which was first developed by Pilowsky in the 1960s. Based on the principle diagnosis criteria of hypochondriasis in DSM IV, the scale consists of 14 items in 3 dimensions: disease phobia, somatic preoccupation and disease conviction (Pilowsky, 1967). Subsequent study has indicated a wide variation of factor models when generalized to different populations (Speckens et al., 1996). Hence, various of derivative of WI were developed, among which the most widely applied version is the 7-item WI (WI-7) developed by Fink et al. *via* latent structure analysis (Fink et al., 1999; Tu et al., 2016; Laferton et al., 2017). Due to the highly overlapped diagnosis criteria of hypochondria and other somatoform disorders, a more specific manifestation was investigated to disentangle hypochondria from somatoform disorder. In an interview study involving 701 participants, it was found that patients with health anxiety generally had the symptom “rumination,” which is not common in patients with somatoform disorder (Fink et al., 2004). Thus, the new item on obsessive rumination, “Recurring thoughts about having a disease that are difficult to get rid of,” was added to the original WI-7 (Carstensen et al., 2020). The updated 8-item (WI-8) may be more helpful in screening for health anxiety.

The aim of the present study was to examine the psychometric properties of an updated WI-8 version to better identify health anxiety in outpatients in general hospitals in China.

## Methods

### Study Design and Setting

Data were derived from a multicenter cross-sectional study. The study sites were three medical settings, departments of traditional Chinese medicine (TCM), neurology/cardiology/gastroenterology (Biomedicine), and psychosomatic medicine (PSY), from nine tertiary grade A hospitals in Beijing, Kunming, Shanghai and Chengdu. Participants who had signed an informed consent were asked to complete questionnaires on sociodemographic information, psychological data, and other clinical characteristics. Afterwards, IAD and SSD were diagnosed using the Structured Clinical Interview for DSM-V (Research Version) (SCDI-5), a semistructured tool for the diagnosis of mental disorders from the DSM-5. To ensure consistency, all interviewers were mental health professionals familiar with DSM-5 classification and passed interview techniques training. They were blinded to the patients questionnaire results. The study was approved by the ethics committees of Peking Union Medical College Hospital (PUMCH) and the University Medical Centre, Freiburg, Germany (Protocol Number: S-K276).

### Patient Recruitment

From May 2016 to March 2017, participants were consecutively recruited from TCM, Biomedicine, and PSY settings on randomly assigned screening days. Participation in the study was voluntary. All the participants endorsed informed consent.

The inclusion criteria were (1) being at least 18 years of age, (2) having sufficient reading and writing skills, (3) attending the hospital visit to receive care oneself, and (4) signing an informed consent form. The exclusion criteria included the following points: (1) a hospital visit for someone else, (2) communication difficulties or language barriers, (3) cognitive impairment, organic brain disorder, dementia, (4) psychosis, or (5) acute suicidal tendency.

**Figure 1:**
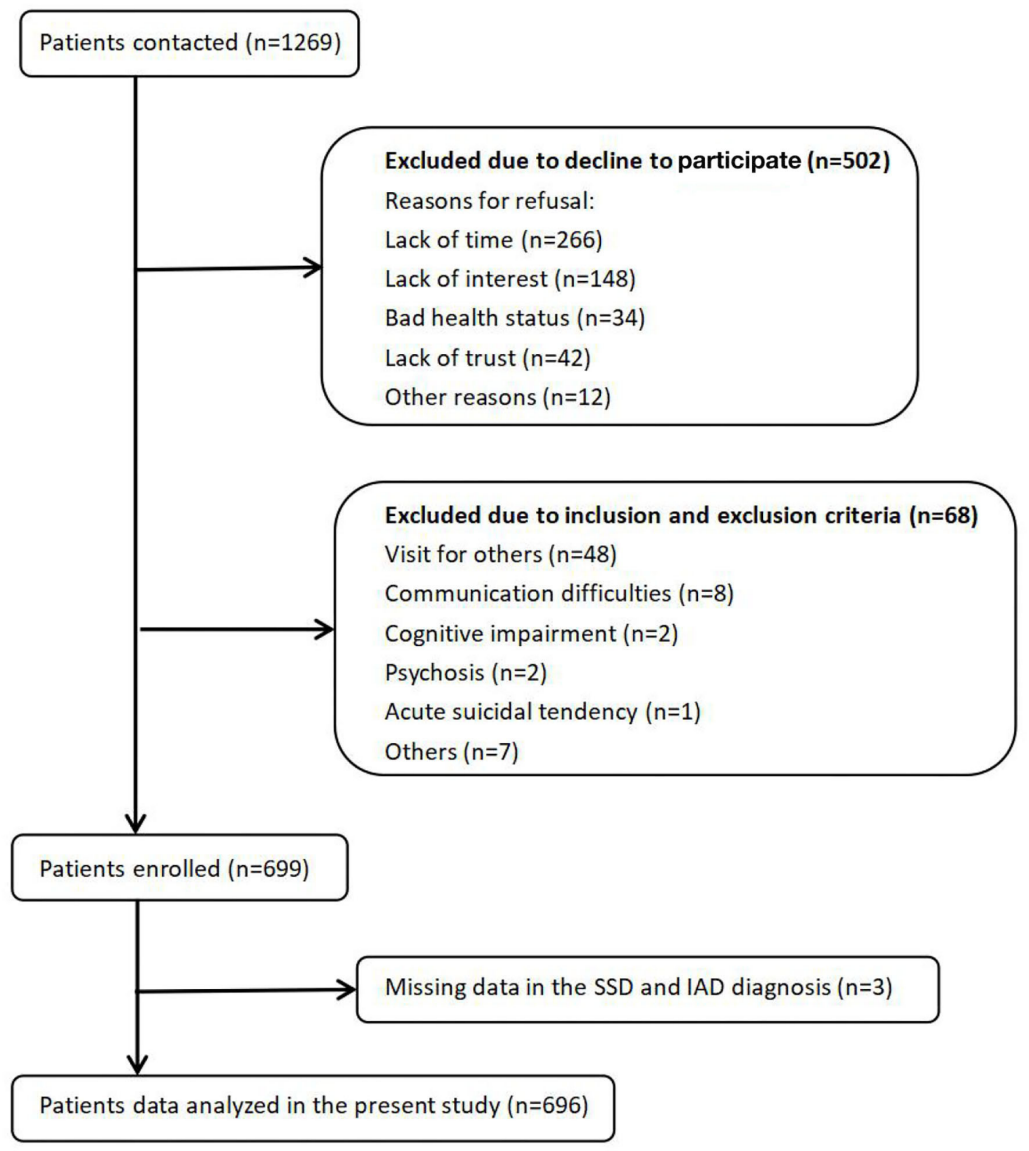
Flow chart of patient enrollment.

Among 1,269 eligible patients, 502 patients refused to participate, 68 patients were excluded on the basis of the exclusion criteria, and 3 additional patients had missing WI-8 score data; therefore, a total of 696 patients were included in this study (see Figure 1).

### Assessment Instruments

#### Whiteley-8 (WI-8)

The WI-8 is a self-reported scale that indicates the severity of health anxiety experienced over the previous 4 weeks. Each item is scored on a scale ranging from 1 to 5 (1 = “no,” 2 = “a little,” 3 = “some,” 4 = “often,” 5 = “severe”). Fink et al. through a large number of interviews found that rumination could better distinguish health anxiety from other somatoform disorders, and therefore proposed this new WI version (Fink et al., 2004; Carstensen et al., 2020). Compared to the well-validated WI-7 (Lee et al., 2011; Tu et al., 2016; Laferton et al., 2017), the WI-8 extends an item, “Recurring thoughts about having a disease that are difficult to get rid of,” to further detect obsessive rumination.

#### Additional Questionnaires

It is well-known that health anxiety commonly co-occurs with somatic symptoms, general anxiety, and depressive symptoms (Kroenke and Rosmalen, 2006). Moreover, these symptoms have distinguishing and additive effects on health-related quality of life, functional status, disability, and health care use (Spitzer et al., 1995; Kroenke et al., 2002; Löwe et al., 2008). Thus, we selected four additional questionnaires, the General Anxiety Disorder (GAD-7), Patient Health Questionnaire-9 (PHQ-9), Patient Health Questionnaire-15 (PHQ-15), and Somatic Symptom Disorder B-criteria (SSD-12), to measure general anxiety, depression and physical symptoms.

(1) General Anxiety Disorder (GAD-7)

The GAD-7 is a brief, self-administered tool to screen for and estimate the severity of generalized anxiety disorders. The GAD-7 contains 7 items, and each item is scored on a scale ranging from 0 to 3. Previous studies have reported that the scale has satisfactory reliability as well as factorial and concurrent validity (He et al., 2010).

(2) Patient Health Questionnaire-9 (PHQ-9)

The PHQ-9 is a 9-item self-report scale that is widely used to screen for depression. The scoring of each item ranges from 0 to 3. The validity and reliability of this scale were confirmed in previous studies (Ran et al., 2017).

(3) Patient Health Questionnaire-15 (PHQ-15)

The PHQ-15 is a self-report questionnaire assessing somatic burden, including the 15 most typical somatic complaints in primary care. Each of the items is rated on a 3-point Likert scale ranging from 0 to 2. This questionnaire has also shown good reliability and validity (Zhang et al., 2016).

(4) Somatic Symptom Disorder B-criteria (SSD-12)

The SSD-12 is a 12-item self-report scale for the psychological criteria of SSD. Each of the items is rated on a 5-point Likert scale ranging from 0 to 5. The reliability and validity of this instrument have been verified in another study (Hüsing et al., 2018; Li et al., 2020).

### Translation of the Questionnaires

The WI-8 was translated and back-translated from English into Chinese using a state-of-the-art procedure for test translation following the “ITC-Test Adaptation Guidelines” (version 2000) of the International Test Commission (ITC) (Merenda, 2006).

The Chinese versions of the PHQ-9, PHQ-15, GAD-7, and SSD-12 have been validated in Chinese samples and published in research papers (He et al., 2010; Zhang et al., 2016; Ran et al., 2017; Li et al., 2020). The questionnaire in Chinese language can be requested from the corresponding author.

### Statistical Analysis

Data were analyzed using IBM SPSS Statistics 24 software and the web-based data science algorithm platform tool SPSSAU. Descriptive data that conformed to the normal distribution were expressed as mean ± standard deviation, and assessed using the *t*-test; non-normally distributed data were expressed as median (quartile spacing), and assessed using the Mann-Whitney-U test. Qualitative variables were expressed as the frequency and percentage, and analyzed using Chi-square test. Given that IAD and SSD share the same characteristics in terms of health anxiety, 233 SSD patients, 12 IAD patients and 3 patients with both diagnoses were classified into the HA group (*n* = 248); the remaining patients constituted the non-HA group (*n* = 448).

The internal consistency of the WI-8 was assessed by Cronbach's alpha coefficient. The convergent and discriminant validity was evaluated based on Kendall's tau-b correlation analysis, since the scores of the scales do not satisfy the normal distribution. Exploratory factor analysis (EFA) was conducted to explore a factor structure of the WI-8. Confirmatory factor analysis (CFA) was performed to evaluate the effect of the new item-8 on the construct validity of WI. The results of CFA were examined according to Hu and Bentler's suggestion that standardized root mean squared residual (SRMR) values <0.08, root mean squared error of approximation (RMSEA) values <0.06, and Comparative Fit Index (CFI) values higher than 0.95 are representative of a close fit of the model (Hu and Bentler, 1999). The diagnostic accuracy was evaluated by receiver operating characteristic (ROC) curve analysis with the area under the curve (AUC).

Statistical significance was assessed at 0.01 alpha level.

## Results

### Sample Characteristics

The present study consisted of 696 patients (61.2% female, 38.8% male, age 43 ± 14.5 years). The patients were predominantly urban dwellers (72.7%), did not live alone (90.7%), and were well-educated (70.0%). However, the HA and non-HA groups did not differ significantly in terms of sociodemographic characteristics (see Table 1).

**Table 1:** Sociodemographic information and clinical characteristics of the study sample (*n* = 696).

	**Total (*n* = 696)**	**Non-HA (*n* = 448)**	**HA (*n* = 248)**	***P***
**Sociodemographic information**				
Age	43 (23)	43 (22)	42 (23)	0.918
Weight/kg	60 (15)	60 (15)	59 (17)	0.085
Gender (man)	270 (38.8%)	171 (38.2%)	99 (39.9%)	0.650
Gender (woman)	426 (61.2%)	277 (61.8%)	149 (60.1%)	
Race (Han)	647 (93.0%)	418 (93.3%)	229 (92.3%)	0.634
Married	506 (72.7%)	335 (74.8%)	171 (69.0%)	0.099
Living situation (city)	572 (82.3%)	374 (83.5%)	198 (80.2%)	0.272
Not living alone	632 (90.8%)	406 (90.6%)	226 (90.7%)	0.826
Income higher than 8,000 yuan per month	216 (31.3%)	145 (32.6%)	71 (28.9%)	0.312
Employed	342 (49.1%)	233 (52.0%)	109 (44.0%)	0.042
High school or higher	514 (73.9%)	340 (75.9%)	174 (70.2%)	0.099
**Clinical characteristics**				
Department				<0.001
Biomedicine	224 (32.2%)	133 (29.7%)	91 (36.7%)	
TCM	230 (33.0%)	182 (40.6%)	48 (19.4%)	
PSY	242 (34.8%)	133 (29.7%)	109 (44.0%)	
Smoking currently	100 (14.4%)	60 (13.4%)	40 (16.2%)	0.314
Alcohol everyday	18 (2.6%)	12 (2.7%)	6 (2.4%)	0.843
Physical inactivity	565 (81.2%)	361 (80.6%)	204 (82.3%)	0.588
Whiteley-8	16 (11)	14 (8)	23 (13)	<0.001
GAD-7	5 (8)	4 (6)	9 (9)	<0.001
PHQ-9	7 (9)	5 (8)	11 (11)	<0.001
PHQ-15	9 (7)	7 (6)	12 (8)	<0.001
SSD-12	11 (20)	6 (13)	24 (16)	<0.001

*HA, health anxiety group (patients with illness anxiety disorder or somatic symptom disorder); Biomedicine, departments of neurology/cardiology/gastroenterology; TCM, department of traditional Chinese medicine; PSY, department of psychosomatic medicine; GAD-7, General Anxiety Disorder; PHQ-9, Patient Health Questionnaire-9; PHQ-15, Patient Health Questionnaire-15; SSD-12, Somatic Symptom Disorder B-criteria*.

### Psychological Features

The mean total score on the WI-8 was 18.26 ± 8.36, with a median score of 16, indicating a positive skewed distribution. The minimum value of the total score was 8, suggesting that almost all the patients were experiencing health anxiety. Patients in the HA group also reported significantly higher GAD-7, PHQ-9, PHQ-15, and SSD-12 scores compared to the non-HA group, suggesting that severe health anxiety is associated with general anxiety, depression, physical symptoms and SSD (see Table 1).

### Reliability

Internal consistency was analyzed with Cronbach's alpha for the total scale score. Cronbach's alpha for the WI-8 was 0.937, and it decreased (0.925) after deleting the 8th item. For the GAD-7, PHQ-9, PHQ-15, and SSD-12, the values were 0.936, 0.893, 0.810, and 0.954, respectively, which also indicated acceptable reliability.

### Factor Analysis

The construct validity of the questionnaire was evaluated by EFA and CFA. To determine the appropriateness of the factor analysis, the sample adequacy for extraction of the factors was confirmed through the Kaiser-Meyer-Olkin (KMO) test (KMO = 0.922) and Bartlett's test of sphericity (*p* < 0.001).

The EFA results revealed that one factor with an eigenvalue over 1 was extracted, accounting for 69.2% of the variance, and the factor loading ranged from 0.688 to 0.901, indicating good construct validity of the 8-item WI.

In the case of modification indices (MI) > 10, the CFA of Fink's two-factor model of the original 6-item WI (Fink et al., 1999) showed that the model fit our data well (RMSEA = 0.056, SRMR = 0.012, CFI = 0.997); While the one-factor 8-item model also showed satisfactory data fit (RMSEA = 0.057, SRMR = 0.019, CFI = 0.993). In addition, the average variance extracted (AVE) was 0.681, and the combined reliability (CR) was 0.943 (see Tables 2, 3).

**Table 2:** Distributions and factor loadings of the WI-8.

**Item**	**Mean (SD)**	**Min**	**Max**	**Factor 1**
1	2.53 (1.26)	1	5	0.857
2	2.36 (1.30)	1	5	0.885
3	2.21 (1.21)	1	5	0.688
4	2.28 (1.33)	1	5	0.901
5	2.30 (1.27)	1	5	0.791
6	1.76 (1.07)	1	5	0.761
7	2.57 (1.22)	1	5	0.861
8	2.24 (1.36)	1	5	0.887

**Table 3:** Goodness-of-fit indices of the model for the Whiteley Index (*n* = 348).

**Model**	**Factors**	**Items**	***X^**2**^/df***	**RMSEA**	**SRMR**	**CFI**
^a*^	1	8	2.131	0.057	0.019	0.993
^b*^	2	6	2.084	0.056	0.012	0.997

a* *Current study*.

b* *Fink et al. (2004)*.

*CFI, Comparative Fit Index; SRMR, standardized root mean squared residual; RMSEA, root mean squared error of approximation*.

### Discriminant Validity

To examine the discriminant validity of the scale, Kendall's tau-b was used to study the relations among the WI-8, GAD-7, PHQ-9, PHQ-15, and SSD-12 scores. The results in Table 4 show that the WI-8 score was moderately positively correlated with the GAD-7 (0.535), PHQ-9 (0.512), PHQ-15 (0.414), and SSD-12 (0.660) scores.

**Table 4:** Correlation coefficients between the WI-8, GAD-7, PHQ-9, PHQ-15, and SSD-12.

	**WI-8**	**GAD-7**	**PHQ-9**	**PHQ-15**
WI-8				
GAD-7	0.535^*^			
PHQ-9	0.512^*^	0.614^*^		
PHQ-15	0.414^*^	0.394^*^	0.463^*^	
SSD-12	0.660^*^	0.492^*^	0.502^*^	0.392^*^

* *P < 0.001*.

### Receiver Operating Characteristic Analyses

**Figure 2:**
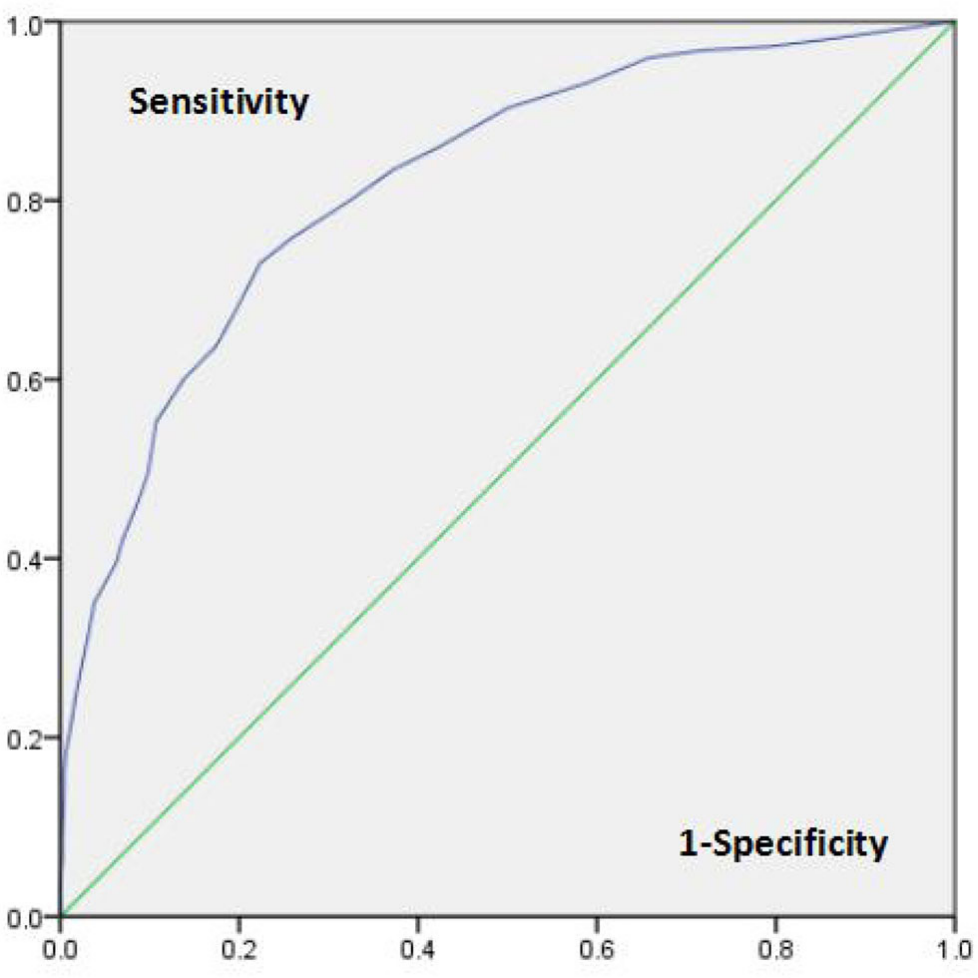
ROC curve of the ability of the WI-8 to distinguish patients with IAD or SSD.

ROC curve analysis was performed to assess the ability of the WI-8 to distinguish participants with health anxiety (IAD patients or SSD patients in our study). The WI-8 showed excellent discriminatory ability, with an AUC of 0.822 (*p* < 0.001, CI = 0.789–0.854). The highest diagnostic accuracy for the WI-8 was achieved by a cutoff value of 19 or higher. The sensitivity and specificity at an optimal cutoff point of 0/1 were 0.730 and 0.777, respectively (see Figure 2).

## Discussion

WI-8 was first developed by Fink et al. (2004) and Carstensen et al. (2020), and our study is the first to perform a psychometric evaluation of the Chinese version of the WI-8 as a health anxiety screening scale. Participants were outpatients in general hospitals, predominantly city dwellers, well-educated and not living alone. The results revealed good reliability, validity and discriminant ability, suggesting that the WI-8 could be used as an effective tool for screening health anxiety.

Cronbach's alpha of the WI-8 (0.937) was higher than that of all the previous versions (between 0.68 and 0.836) (Pilowsky, 1967; Welch et al., 2009; Lee et al., 2011; Hedman et al., 2015; Fergus et al., 2018). Moreover, Cronbach's alpha decreased after deleting the 8th item, indicating that the additional item 8 is not redundant, but increased the consistency of the scale. Various studies have reported the factor structure of WI and proposed unitary, 2-factor, and 3-factor models consisting of 6–14 items, indicating that the internal structure is unstable (Pilowsky, 1967; Welch et al., 2009). Therefore, the entire sample was randomly split into two parts: one group (*n* = 348) explores the WI-8 factor structure by EFA, the other group (*n* = 348) compares the goodness of fit of different factor structures by CFA. EFA indicated a single factor model for WI-8, suggesting that the newly added item did not alter the one-factor structure of the original WI-7. Previous studies have also proposed that the original three factors of WI are highly related, and the one-factor model may be more suitable (Speckens et al., 1996). While Fink et al. (2004) pointed out that two isolated factors were more consistent with clinical characteristics, and they proposed a two-factor model including Illness Conviction subscale and Illness Worrying subscale. However, we compared the fitting index of the two models and found that the two-factor model was not significantly better than the single-factor model. Furthermore, the AVE and CR indicated good convergent validity. It is possible that participants tended to rate all questions on the questionnaire consistently, resulting in a single structure.

The WI-8 had moderate correlations with the GAD-7, PHQ-9, and PHQ-15, whereas in a study involving 200 patients and 240 healthy graduate students, the WI-7 had lower correlations with the GAD-7 (0.35), PHQ-9 (0.30), and PHQ-15 (0.33) (Güleç et al., 2012), perhaps because these psychological factors were more closely linked in patients than in healthy people. Furthermore, the WI-8 had the weakest correlation with the PHQ-15 and the strongest correlation with the SSD-12. These findings are consistent with the IAD characteristics of light somatic symptoms and excessive health-related thoughts, and also suggest that health anxiety is an important feature of SSD.

The WI-8 showed excellent discriminatory ability to distinguish patients with health anxiety, which is consistent with WI-6 (AUC = 0.83) (Fergus et al., 2019), and superior to WI-7 (AUC = 0.660) (Tu et al., 2016). The results indicated that the WI-8 offers a better way to identify persons with health anxiety, likely because of the additional item emphasizing recurring thoughts.

This study has several limitations. The stability of the WI-8 over time could not be evaluated due to the cross-sectional study design. Additionally, the self-report scale could have been influenced by the educational background of the participants. Moreover, too few IAD patients could be analyzed as a subgroup, which affected the testing of the discriminant ability of the WI-8. Therefore, we grouped IAD patients and SSD patients into one group based on the similarity of their health anxiety characteristics. A lower proportion of IAD patients may be due to their mild physical symptoms and less hospital visits. Hence, future research with appropriate sample is planned to confirm and extend our findings in patients with general anxiety.

## Conclusion

The latest version of the 8-item WI has satisfactory reliability, validity and discriminant ability in general hospital outpatients in China. Therefore, we introduce the WI-8 as an assessment tool for screening health anxiety.

## Data Availability Statement

The raw data supporting the conclusions of this article will be made available by the authors, without undue reservation.

## Ethics Statement

The studies involving human participants were reviewed and approved by the ethics committees of Peking Union Medical College Hospital (PUMCH) and the University Medical Centre, Freiburg, Germany (Protocol Number: S-K276). The patients/participants provided their written informed consent to participate in this study.

## Author Contributions

HW, KF, PF, JW, LZ, and WLi contributed conception and design of the study. YZ, HC, XM, JR, and WLu contributed to data collection. YC and RL performed the statistical analysis. YC wrote the first draft of the manuscript. HW, KF, RL, and A-KT wrote sections of the manuscript. All authors contributed to manuscript revision, read, and approved the submitted vision.

## Conflict of Interest

JR was employed by General Hospital of Jincheng Anthracite Coal Mining Group Co. Ltd., Jincheng, China. The remaining authors declare that the research was conducted in the absence of any commercial or financial relationships that could be construed as a potential conflict of interest.

## Abbreviations

DSM-IV: Diagnostic and Statistical Manual of Mental Disorder (4th Edition)
DSM-V: Diagnostic and Statistical Manual of Mental Disorder (5th Edition)
GAD-7: General Anxiety Disorder
IAD: Illness Anxiety Disorder
PHQ-9: Patient Health Questionnaire-9
PHQ-15: Patient Health Questionnaire-15
ROC: Receiver Operating Characteristic
SSD: Somatic Symptom Disorder
SSD-12: Somatic Symptom Disorder B-criteria
WI: Whiteley Index.
